# A Rare Case of PCH10 With CLP1 Mutation: Clinical Features and Dental Management of Riga–Fede Lesion in a Pediatric Patient

**DOI:** 10.1155/crid/9306930

**Published:** 2026-10-09

**Authors:** Merve Mısır, Yelda Kasimoglu, Mine Koruyucu

**Affiliations:** ^1^ Department of Pedodontics Faculty of Dentistry Istanbul University Istanbul Turkey istanbul.edu.tr

**Keywords:** CLP1 mutation, pediatric dentistry, pontocerebellar hypoplasia Type 10, Riga–Fede disease

## Abstract

**Background:**

Pontocerebellar hypoplasia Type 10 (PCH10) is an extremely rare autosomal recessive neurodegenerative disorder caused by biallelic mutations in the *CLP1* gene. The disease is characterized by severe developmental delay, progressive microcephaly, epilepsy, spasticity, and craniofacial dysmorphic features. To our knowledge, Riga–Fede disease in a patient with genetically confirmed PCH10 has not been previously reported.

**Case Presentation:**

We report the case of a 21‐month‐old girl with genetically confirmed PCH10 who presented with severe feeding difficulties and a ventral tongue ulcer caused by repeated trauma from the mandibular central primary incisors. The lesion was diagnosed as Riga–Fede disease. Because of persistent trauma, extraction of the offending teeth was performed under local anesthesia, which resulted in complete healing within follow‐up. The patient′s clinical course was further complicated by epilepsy, profound hypotonia, lack of motor control, and recurrent respiratory infections, requiring percutaneous endoscopic gastrostomy (PEG) feeding and intensive care admissions. Family history revealed the death of a sibling with a similar clinical presentation, supporting autosomal recessive inheritance.

**Conclusion:**

This case highlights the multisystemic burden of PCH10 and emphasizes the importance of early recognition and management of oral complications such as Riga–Fede disease. Multidisciplinary care, including neurological, genetic, and dental follow‐up, is critical for improving the quality of life in children with rare neurodegenerative disorders.

## 1. Introduction

Pontocerebellar hypoplasia Type 10 (PCH10) (OMIM#615803) is a rare genetic disorder caused by biallelic mutations in the *CLP1* gene. This condition is characterized by structural abnormalities of the brain, progressive microcephaly, and severe intellectual and motor impairments, as well as clinical features such as spasticity [1, 2]. The *CLP1* gene, located in the chromosomal region 1q12.1, was first described by Weitzer and Martinez in 2007 [3]. This gene is responsible for encoding various multifunctional kinase proteins, including the tRNA splicing endonuclease (TSEN) complex and the pre‐mRNA cleavage Complex II [1]. *CLP1* plays a critical role particularly in the processes of tRNA formation and maturation [3]. During biosynthetic processes, tRNAs undergo a series of posttranscriptional modifications as they transition from their pre‐tRNA form to the active tRNA form [4].

Mutations in *CLP1* and in the four subunits of the TSEN complex (*TSEN54*, *TSEN2*, *TSEN34*, and *TSEN15*) have been associated with various neurodegenerative disorders characterized by pontocerebellar hypoplasia (PCH) [4]. The clinical manifestations and neuroimaging findings in these disorders may vary, and involvement of supratentorial structures can also accompany the presentation [5–7]. The first case associated with PCH was reported in the literature in 1917. Subsequently, Barth et al. [8] diagnosed PCH in seven children from five families and described the clinical features of the disease. In the reported cases, involuntary motor movements, extrapyramidal system disorders, and microcephaly were the prominent findings. Barth et al. also made the first subtype distinction for PCH, classifying the disease into PCH Type 1 and PCH Type 2. PCH Type 1 is generally characterized by muscle weakness and hypotonia resulting from degeneration of anterior horn motor neurons. In contrast, PCH Type 2 is marked by cognitive and neurodevelopmental impairment, typically presenting in the neonatal period with symptoms such as jitteriness, poor sucking and swallowing abilities, chorea, or dyskinesias. The clinical features of PCH were first described in detail by Krause in 1928 [9]. Krause described a 16‐month‐old child with an atrophic appearance who presented with swallowing difficulties, muscle spasms, and sudden involuntary movements of the arms and legs. Retrospective evaluations conducted in later years suggest that this description is consistent with PCH Type 2.

Subsequently, in 1961, the first case demonstrating spinal motor neuron degeneration in association with PCH was reported in the literature. Clinically, this presentation resembled Werdnig–Hoffmann disease, also known as spinal muscular atrophy (SMA) [10]. In the following years, similar reported cases demonstrated that the coexistence of pontine and cerebellar hypoplasia with bulbospinal motor neuron involvement represented a distinct clinical syndrome rather than a coincidental association [11–13].

Riga–Fede disease is a traumatic ulcerative lesion affecting the ventral surface of the tongue as a result of repetitive mechanical trauma, most commonly from newly erupted mandibular incisors. Although it is generally associated with natal or neonatal teeth, it may also occur after eruption of the primary incisors and can interfere with feeding if left untreated. Importantly, previous reports have emphasized that Riga–Fede disease may represent the first clinical manifestation of underlying neurological disorders associated with abnormal tongue movements or impaired oral motor control, including cerebral palsy, familial dysautonomia, Lesch–Nyhan syndrome, and microcephaly [14, 15].

## 2. Case Report

On December 21, 2023, a 1.5‐year‐old patient was admitted to the Department of Pediatric Dentistry, Faculty of Dentistry, Istanbul University, with the complaint of “ulceration in the sublingual region and feeding difficulties.” The systemic medical history revealed a diagnosis of PCH10 due to a *CLP1* mutation. The systemic medical history indicated that the patient had previously been diagnosed with PCH10 associated with a *CLP1* mutation, as reported by her treating physician. However, the original genetic test report and neuroimaging findings were not available to the authors for independent review. The patient presented with profound generalized hypotonia and was unable to achieve head or trunk control. She was completely dependent on caregiver support for positioning and transportation and was brought to the clinic in a stroller. No meaningful motor milestones had been achieved, and severe impairment of oral motor function was evident during the clinical examination, with marked feeding difficulties and poor tongue control. The patient had a history of epilepsy and was under regular follow‐up by pediatric neurology and medical genetics services. Current medications included pantoprazole as a gastric protector, and baclofen, clobazam, phenobarbital, topiramate, and valproic acid for seizure management.

Extraoral examination revealed hypertelorism, a smooth philtrum, a short neck, and lateral deviation of the eyes (Figure 1). Intraoral examination revealed a well‐defined traumatic ulcer located on the ventral surface of the tongue, directly opposing the mandibular primary central incisors (71 and 81). The lesion was consistent with Riga–Fede disease and was associated with continuous mechanical irritation during involuntary tongue movements. The persistent ulceration caused significant discomfort during oral feeding. (Figure 2). Although no mobility was detected in the teeth, the trauma persisted and was significantly interfering with the patient′s oral feeding. The parents reported that the patient would soon require percutaneous endoscopic gastrostomy (PEG) feeding.

**Figure 1:**
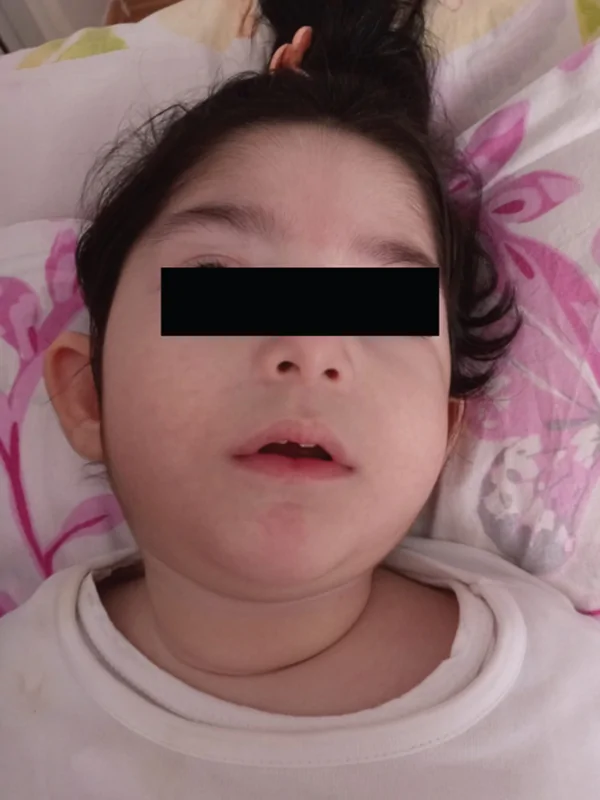
Extraoral photography of the patient.

**Figure 2:**
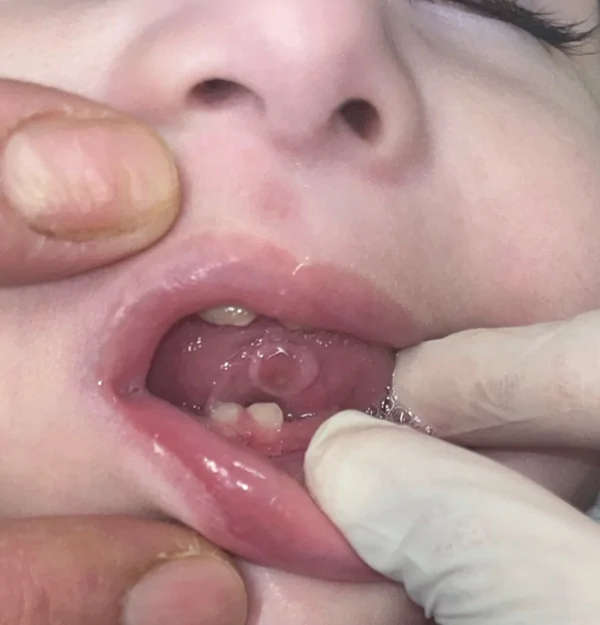
Riga–Fede lesion observed on the ventral surface of the tongue caused by the primary Teeth 71 and 81.

To prevent progression of the Riga–Fede ulceration, Teeth 71 and 81 were extracted under local anesthesia. A soft diet was recommended, and regular follow‐up visits were scheduled.

At the follow‐up examination at 25 months of age, complete healing of the Riga–Fede ulceration and resolution of oral trauma were observed (Figure 3). During the follow‐up period, on August 8, 2025, the patient developed a severe pulmonary infection, with the bronchi filled with saliva and infected secretions. As a result, the patient was admitted to the intensive care unit, intubated, and fed via PEG. No dental complications were reported during this period.

**Figure 3:**
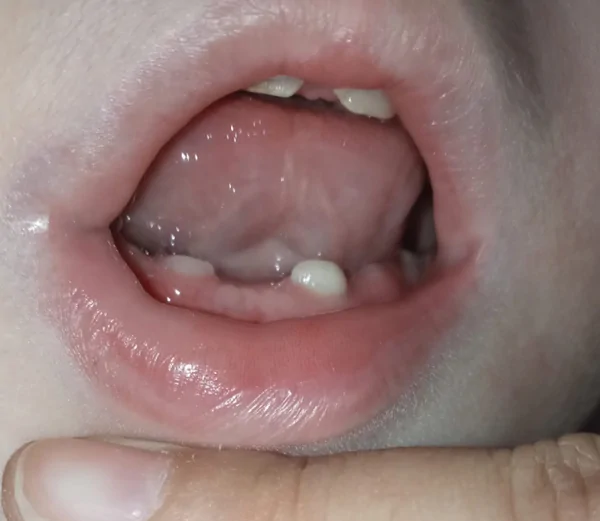
Healed appearance of the Riga–Fede lesion.

The family pedigree is shown in Figure 4, demonstrating autosomal recessive inheritance with two affected children. Family history revealed that a previously born sibling had presented with a similar clinical picture and had died because of infections and feeding difficulties. No similar genetic or clinical findings were reported in other family members. During the follow‐up period, the mother was pregnant. At the last control examination, performed when the patient was 40 months old, the family reported that the newborn sibling was healthy and exhibiting normal developmental progress.

**Figure 4:**
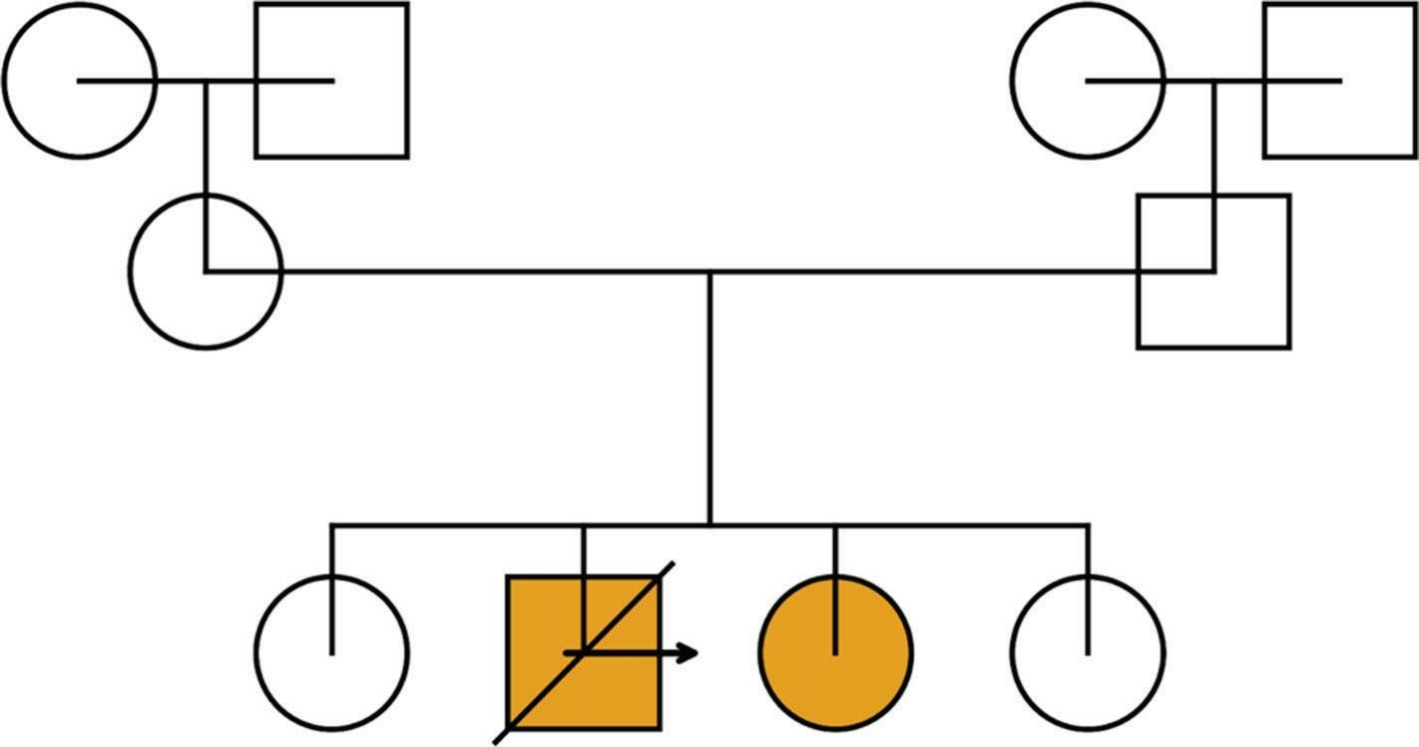
Family pedigree.

## 3. Discussion

Several studies have reported that PCH10 caused by *CLP1* mutations is characterized by severe developmental delay, epilepsy, progressive microcephaly, hypotonia or spasticity, and characteristic craniofacial dysmorphism. Neuroimaging findings commonly include PCH, delayed myelination, and brainstem abnormalities. Consistent with previous reports, our patient presented with profound hypotonia, epilepsy, feeding difficulties, craniofacial dysmorphic features, and severe motor impairment, reflecting the characteristic clinical phenotype of PCH10 [1, 2].

Published reports have consistently described PCH10 as a severe neurodevelopmental disorder characterized by developmental delay, epilepsy, progressive microcephaly, feeding difficulties, hypotonia, and characteristic craniofacial dysmorphism. Wafik et al. [16] further expanded the clinical spectrum by reporting additional Turkish patients with genetically confirmed PCH10. Consistent with these reports, our patient presented with profound hypotonia, epilepsy, severe motor impairment, feeding difficulties, and craniofacial dysmorphic features, supporting the characteristic phenotype of PCH10. Previous studies describing *CLP1* mutation‐associated PCH10 and other PCH subtypes are summarized in Table 1.

**Table 1:** Summary of studies describing CLP1 mutation‐associated PCH10 and other PCH subtypes.

Author/year	Study group/case	Genetic findings	Clinical features	Key findings/contributions
Karaca et al., 2014 [1]	Cases with CLP1 mutation	CLP1 mutation → impaired tRNA biogenesis	Peripheral + CNS involvement, microcephaly, epilepsy	Defined the role of CLP1 in tRNA biology
Schaffer et al., 2014 [2]	Turkish families	CLP1 founder mutation	Microcephaly, spasticity, epilepsy, motor and sensory neuropathy	Expanded PCH10 spectrum; MRI: thin corpus callosum, pontine hypoplasia
Wafik et al., 2018 [16]	Two new Turkish cases	Homozygous missense CLP1 mutation	Developmental delay, epilepsy, microcephaly	Additional families described
Van Dijk et al., 2018 [5]	Review on PCH genes	TSEN complex and CLP1 association	Genetic basis of PCH subtypes	Updated classification of genetic/clinical subtypes
Nuovo et al., 2022 [6]	Multicenter study	TSEN genes + CLP1 mutations	Wide phenotypic variability	Broadened genotype–phenotype correlations
Namavar et al., 2011 [7]	Various PCH cases	TSEN and CLP1 association	Microcephaly, epilepsy, developmental delay	Systematic clinical and neuroradiological description
Barth et al., 1990 [8]	seven children, five families	Defined PCH1 and PCH2	Microcephaly, dyskinetic movements, hypotonia	First clinical classification of PCH
Krause, 1929 [9]	Single case	—	Dysphagia, spasms, involuntary movements	Earliest detailed clinical description
Norman, 1961 [10]	Single case	—	SMA‐like phenotype + cerebellar hypoplasia	Differentiation from SMA
Goutières and Aicardi, 1977 [11]	Infant cases	—	Anterior horn cell involvement + PCH	Association with motor neuron disease

The present case highlights the clinical course and multisystemic complications of a rare pediatric neurological disorder diagnosed as PCH10 due to a *CLP1* gene mutation. The patient′s neurological findings, including severe muscular hypotonia, epilepsy, and loss of motor control, reflect the clinical profile of PCH10 as described in the literature. Furthermore, complications such as feeding difficulties and recurrent pulmonary infections emphasize the systemic impact commonly observed in neurodegenerative disorders of this type. The family history of a previously affected child with a similar clinical presentation underscores the genetic transmission and the severe nature of the disease.

There is currently no disease‐modifying treatment for PCH, and management remains largely supportive and multidisciplinary. Feeding difficulties, epilepsy, respiratory complications, and motor impairment are among the major clinical challenges requiring individualized care. In the present case, symptomatic management included antiepileptic therapy, nutritional support with PEG, and regular neurological, genetic, and dental follow‐up, reflecting the multidisciplinary approach recommended for children with severe neurodevelopmental disorders [17]. The present case highlights the importance of multidisciplinary care in children with PCH10. Management required close collaboration between pediatric neurologists, medical geneticists, pediatric dentists, and other healthcare professionals to address feeding difficulties, epilepsy, and oral complications. This case underscores the need for comprehensive clinical evaluation and coordinated follow‐up to optimize the quality of life of children with rare neurodevelopmental disorders.

Riga–Fede disease is generally regarded as a traumatic ulcerative lesion resulting from repetitive injury of the ventral tongue. Although the lesion is benign, delayed diagnosis may interfere with feeding and lead to dehydration, poor nutritional intake, and additional medical complications. Therefore, early recognition and elimination of the traumatic source are essential. Baghdadi subsequently described Riga–Fede disease in an infant with microcephaly and highlighted that neurological impairment and abnormal tongue movements may predispose affected children to repetitive lingual trauma. In that case, conservative management consisting of smoothing the incisal edges and topical triamcinolone resulted in complete healing [15].

Similar to that report, our patient also had severe neurological impairment; however, unlike isolated microcephaly, she had genetically confirmed PCH10 caused by a *CLP1* mutation, accompanied by profound hypotonia, epilepsy, loss of head and trunk control, and severe feeding difficulties. These neurological manifestations likely increased repetitive tongue trauma and contributed to the development of Riga–Fede disease.

In this patient, the ulcerative lesion on the ventral surface of the tongue was attributed to repetitive trauma from the mandibular primary central incisors (Teeth 71 and 81). The patient′s profound muscular hypotonia, lack of head and trunk control, and recurrent epileptic seizures likely resulted in impaired oral motor control and repetitive tongue movements, leading to chronic mechanical trauma and progression of the ulceration.

Previous reports recommend conservative treatment as the initial approach, including smoothing of incisal edges, topical corticosteroids, composite resin coverage, or protective appliances, whereas extraction should be reserved for persistent lesions or situations in which conservative measures are unlikely to succeed [14, 15].

In the present patient, extraction was considered the most appropriate treatment because persistent tongue trauma occurred in the setting of profound hypotonia, recurrent epileptic seizures, absence of head and trunk control, and severe feeding impairment requiring PEG placement. Under these circumstances, conservative treatment was considered unlikely to eliminate repetitive trauma reliably.

Previous reports have described Riga–Fede disease in otherwise healthy infants and in children with neurological conditions such as microcephaly [15]. However, to our knowledge, Riga–Fede disease has not previously been reported in a child with genetically confirmed PCH10 caused by a *CLP1* mutation. This case therefore expands the spectrum of neurological disorders associated with Riga–Fede disease and highlights the importance of early dental assessment in children with severe neurodevelopmental impairment.

## 4. Conclusion

This case highlights the importance of early recognition and appropriate management of Riga–Fede disease in children with severe neurodevelopmental disorders such as PCH10. In patients with profound hypotonia, impaired oral motor control, and feeding difficulties, persistent traumatic tongue ulceration should prompt timely dental evaluation. When conservative treatment is unlikely to eliminate the source of trauma, extraction of the offending teeth may represent an appropriate therapeutic option. Early multidisciplinary collaboration between pediatric dentists, neurologists, and geneticists is essential to improve oral function, facilitate feeding, and enhance the quality of life of affected children.

## Author Contributions

Merve Mısır: Conceptualization, investigation, data curation, writing—original draft, and visualization. Yelda Kasimoglu: Investigation, clinical management, and writing—review and editing. Mine Koruyucu: Supervision, validation, and writing—review and editing.

## Funding

No funding was received for this manuscript.

## Disclosure

All authors read and approved the final version of the manuscript.

## Ethics Statement

Ethics approval was not required for this case report, as it describes a single patient and does not involve experimental interventions. Written informed consent for participation was obtained from the patient′s legal guardians.

## Consent

Written informed consent was obtained from the patient′s parents for publication of this case report and the accompanying clinical images. A copy of the consent is available for review by the editor of this journal.

## Conflicts of Interest

The authors declare no conflicts of interest.

## Supporting information


**Supporting Information** Additional supporting information can be found online in the Supporting Information section. File S1: CARE checklist of information to include when writing a case report.

## Data Availability

All data generated or analyzed during this study are included in this published article. No additional datasets were generated. The dataset supporting the conclusions of this article is included within the article.

## References

[1] Karaca E. , Weitzer S. , Pehlivan D. , Shiraishi H. , Gogakos T. , Hanada T. , Jhangiani S. N. , Wiszniewski W. , Withers M. , Campbell I. M. , and Erdin S. , Human CLP1 Mutations Alter tRNA Biogenesis, Affecting Both Peripheral and Central Nervous System Function, Cell. (2014) 157, no. 3, 636–650. 10.1016/j.cell.2014.02.058 PMC4146440 24766809

[2] Schaffer A. E. , Eggens V. R. , Caglayan A. O. , Reuter M. S. , Scott E. , Coufal N. G. , Silhavy J. L. , Xue Y. , Kayserili H. , Yasuno K. , Rosti R. O. , Abdellateef M. , Caglar C. , Kasher P. R. , Cazemier J. L. , Weterman M. A. , Cantagrel V. , Cai N. , Zweier C. , Altunoglu U. , Satkin N. B. , Aktar F. , Tuysuz B. , Yalcinkaya C. , Caksen H. , Bilguvar K. , Fu X. D. , Trotta C. R. , Gabriel S. , Reis A. , Gunel M. , Baas F. , and Gleeson J. G. , CLP1 Founder Mutation Links tRNA Splicing and Maturation to Cerebellar Development and Neurodegeneration, Cell. (2014) 157, no. 3, 651–663, 10.1016/j.cell.2014.03.049, 24766810. PMC4128918 24766810

[3] Weitzer S. and Martinez J. , The Human RNA Kinase hClp1 Is Active on 3 Transfer RNA Exons and Short Interfering RNAs, Nature. (2007) 447, no. 7141, 222–226, 10.1038/nature05777, 17495927. 17495927

[4] Hanada T. , Weitzer S. , Mair B. , Bernreuther C. , Wainger B. J. , Ichida J. , Hanada R. , Orthofer M. , Cronin S. J. , Komnenovic V. , Minis A. , Sato F. , Mimata H. , Yoshimura A. , Tamir I. , Rainer J. , Kofler R. , Yaron A. , Eggan K. C. , Woolf C. J. , Glatzel M. , Herbst R. , Martinez J. , and Penninger J. M. , CLP1 links tRNA Metabolism to Progressive Motor-Neuron Loss, Nature. (2013) 495, no. 7442, 474–480, 10.1038/nature11923, 23474986. PMC3674495 23474986

[5] van Dijk T. , Baas F. , Barth P. G. , and Poll-The B. T. , What′s New in Pontocerebellar Hypoplasia? An Update on Genes and Subtypes, Orphanet Journal of Rare Diseases. (2018) 13, no. 1, 10.1186/s13023-018-0826-2, 29903031. PMC6003036 29903031

[6] Nuovo S. , Micalizzi A. , Romaniello R. , Arrigoni F. , Ginevrino M. , Casella A. , Serpieri V. , D′Arrigo S. , Briguglio M. , Salerno G. G. , Rossato S. , Sartori S. , Leuzzi V. , Battini R. , Ben-Zeev B. , Graziano C. , Mirabelli Badenier M. , Brankovic V. , Nardocci N. , Spiegel R. , Petković Ramadža D. , Vento G. , Marti I. , Simonati A. , Dipresa S. , Freri E. , Mazza T. , Bassi M. T. , Bosco L. , Travaglini L. , Zanni G. , Bertini E. S. , Vanacore N. , Borgatti R. , and Valente E. M. , Refining the Mutational Spectrum And Gene–Phenotype Correlates in Pontocerebellar Hypoplasia: Results of a Multicentric Study, Journal Of Medical Genetics. (2022) 59, no. 4, 399–409, 10.1136/jmedgenet-2020-107497, 34085948. 34085948

[7] Namavar Y. , Barth P. G. , Kasher P. R. , Van Ruissen F. , Brockmann K. , Bernert G. , Writzl K. , Ventura K. , Cheng E. Y. , and Ferriero D. M. , Clinical, Neuroradiological and Genetic Findings in Pontocerebellar Hypoplasia, Brain. (2011) 134, no. 1, 143–156, 10.1093/brain/awq287, 20952379. PMC9136852 20952379

[8] Barth P. , Vrensen G. , Uylings H. , Oorthuys J. , and Stam F. , Inherited Syndrome of Microcephaly, Dyskinesia and Pontocerebellar Hypoplasia: A Systemic Atrophy With Early Onset, Journal Of The Neurological Sciences. (1990) 97, no. 1, 25–42, 10.1016/0022-510X(90)90096-6, 2370559. 2370559

[9] Krause F. , Über einen Bildungsfehler des Kleinhirns und Einige Faseranatomische Beziehungen des Organs, Zeitschrift für die Gesamte Neurologie und Psychiatrie. (1929) 119, no. 1, 788–815, 10.1007/BF02863836.

[10] Norman R. , Cerebellar Hypoplasia in Werdnig-Hoffmann disease, Archives of Disease in Childhood. (1961) 36, no. 185, 96–101, 10.1136/adc.36.185.96, 13729575. PMC2012675 13729575

[11] Goutières F. , Aicardi J. , and Farkas E. , Anterior Horn Cell Disease Associated With Pontocerebellar Hypoplasia in Infants, Journal of Neurology, Neurosurgery & Psychiatry. (1977) 40, no. 4, 370–378, 10.1136/jnnp.40.4.370, 874513. PMC492704 874513

[12] Kamoshita S. , Takei Y. , Miyao M. , Yanagisawa M. , Kobayashi S. , and Saito K. , Pontocerebellar Hypoplasia Associated With Infantile Motor Neuron Disease (Norman′s disease), Pediatric Pathology. (1990) 10, no. 1-2, 133–142, 10.3109/15513819009067102, 2315227. 2315227

[13] Chou S. , Gilbert E. , Chun R. , Laxova R. , Tuffli G. , Sufit R. , and Krassikot N. , Infantile Olivopontocerebellar Atrophy With Spinal Muscular Atrophy (Infantile OPCA+ SMA), Clinical neuropathology. (1990) 9, no. 1, 21–32, 2407400. 2407400

[14] Baghdadi Z. , Riga-Fede Disease: Report of a Case and Review, Journal of Clinical Pediatric Dentistry. (2001) 25, no. 3, 209–213, 10.17796/jcpd.25.3.9725k31q263800km, 12049080. 12049080

[15] Baghdadi Z. , Riga–Fede Disease: Association With Microcephaly, International Journal of Paediatric Dentistry.(2002) 12, no. 6, 442–445, 10.1046/j.1365-263X.2002.00396.x, 12452988. 12452988

[16] Wafik M. , Taylor J. , Lester T. , Gibbons R. J. , and Shears D. J. , 2 New Cases of Pontocerebellar Hypoplasia Type 10 Identified by Whole Exome Sequencing in a Turkish Family, European Journal Of Medical Genetics. (2018) 61, no. 5, 273–279, 10.1016/j.ejmg.2018.01.002, 29307788. 29307788

[17] Sánchez-Albisua I. , Frölich S. , Barth P. G. , Steinlin M. , and Krägeloh-Mann I. , Natural Course of Pontocerebellar Hypoplasia Type 2A, Orphanet Journal of Rare Diseases. (2014) 9, no. 1, 10.1186/1750-1172-9-70, 24886362. PMC4019562 24886362

